# Insertion Sequences within Oxacillinases Genes as Molecular Determinants of *Acinetobacter baumannii* Resistance to Carbapenems—A Pilot Study

**DOI:** 10.3390/microorganisms12102057

**Published:** 2024-10-12

**Authors:** Dagmara Depka, Tomasz Bogiel, Mateusz Rzepka, Eugenia Gospodarek-Komkowska

**Affiliations:** 1 Microbiology Department, Ludwik Rydygier Collegium Medicum in Bydgoszcz, Nicolaus Copernicus University in Torun, 85-094 Bydgoszcz, Poland; dagmaradepka@cm.umk.pl (D.D.); mateusz.rzepka@cm.umk.pl (M.R.); gospodareke@cm.umk.pl (E.G.-K.); 2 Department of Clinical Microbiology, Antoni Jurasz University Hospital No. 1, 85-094 Bydgoszcz, Poland

**Keywords:** *Acinetobacter baumannii*, carbapenem resistance, insertion sequences, molecular basis of antimicrobial resistance, oxacillinases

## Abstract

Carbapenem-resistant *Acinetobacter baumannii* is one of the major problems among hospitalized patients. The presence of multiple virulence factors results in bacteria persistence in the hospital environment. It facilitates bacterial transmission between patients, causing various types of infections, mostly ventilator-associated pneumonia and wound and bloodstream infections. *A. baumannii* has a variable number of resistance mechanisms, but the most commonly produced are carbapenem-hydrolyzing class D β-lactamases (CHDLs). In our study, the presence of *bla*_OXA-23_, *bla*_OXA-40_ and *bla*_OXA-51_ genes was investigated among 88 clinical isolates of *A. baumannii*, including 53 (60.2%) strains resistant to both carbapenems (meropenem and imipenem) and 35 (39.8%) strains susceptible to at least meropenem. Among these bacteria, all the isolates carried the *bla*_OXA-51_ gene. The *bla*_OXA-23_ and *bla*_OXA-40_ genes were detected in two (5.7%) and three (8.6%) strains, respectively. Among the OXA-23 carbapenemase-producing *A. baumannii* strains (*n* = 55), insertion sequences (IS*Aba1*) were detected upstream of the *bla*_OXA-23_ gene in fifty-two (94.5%) carbapenem-resistant and two (3.6%) meropenem-susceptible isolates. *A. baumannii* clinical strains from Poland have a similar antimicrobial resistance profile as those worldwide, with the presence of IS*Aba1* among *bla*_OXA-23_-positive isolates also being quite common. Carbapenem resistance among *A. baumannii* strains is associated with the presence of CHDLs, especially when insertion sequences are present.

## 1. Introduction

*Acinetobacter baumannii* belong to glucose-non-fermentative, non-motile, non-fastidious, aerobic Gram-negative coccobacilli. They are a part of the *Acinetobacter calcoaceticus*–*A. baumannii* complex (Acb, consisting of *A. baumannii*, *A. calcoaceticus*, and *Acinetobacter* genomic species 3 and *Acinetobacter* genomic species 13TU (Tjernberg and Ursing)—two of the most clinically relevant genospecies in the Acb complex), associated with a number of infections in clinical practice [1]. Antimicrobial resistance among Gram-negative rods currently is a major global healthcare threat [2,3]. Carbapenem-resistant *A. baumannii* is one of the most frequently isolated pathogens from hospitalized patients, especially in intensive care units (ICUs) worldwide [4,5].

*A. baumannii* are isolated from various types of infections, mostly ventilator-associated pneumonia (VAP), wounds, skin and soft tissue, urinary tract infections, meningitis and bloodstream infections [6,7,8]. The presence of multiple virulence factors (e.g., biofilm formation) results in bacteria persistence in the hospital environment and facilitates transmission between patients [9,10]. Nosocomial infections caused by *A. baumannii* are also the most prevalent among critically ill patients, as their risk factors include prolonged hospital stays, advanced age, invasive procedure application and mechanical ventilation introduction [11].

The choice of an effective treatment for infections with *A. baumannii* etiology is very difficult. *A. baumannii* rods are naturally resistant to ampicillin, amoxicillin (and its combination with clavulanic acid), cefazolin, cefotaxime, ceftriaxone, aztreonam, ertapenem, trimethoprim and fosfomycin [12]. Hence, *A. baumannii* has been included in the ESKAPE (*Enterococcus faecium*, *Staphylococcus aureus*, *Klebsiella pneumoniae*, *A. baumannii*, *Pseudomonas aeruginosa*, and *Enterobacter* spp.) group of pathogens that has been described to emphasize the ability of these pathogens to avoid antimicrobial effect [13]. Of note, carbapenems (imipenem, meropenem) are often used in empirical therapy when infection with Gram-negative coccobacilli is suspected. According to various reports, up to 90% of *A. baumannii* strains derived from patients with different kinds of infections are unfortunately resistant to carbapenems [4,14].

*A. baumannii* has three main antibiotic resistance mechanisms: (i) control of antimicrobial transportation through bacterial membranes, (ii) modification of antibiotic targets and (iii) enzymatic inactivation of the antimicrobials [3,15]. However, the structure of their cells and the plasticity of the genome additionally enable the acquisition of new resistance mechanisms, e.g., genes encoding enzymes that hydrolyze antibiotics—mostly β-lactamases.

All four classes of β-lactamases (according to Ambler’s classification) have been identified among *A. baumannii* isolates, including a large number of carbapenem-hydrolyzing enzymes (carbapenemases). *A. baumannii* isolates produce various types of carbapenemases: KPC, GES, NDM, VIM, IMP and SIM [6,15]. However, the most common are carbapenem-hydrolyzing class D β-lactamase groups (CHDLs, oxacillinases–OXA). The major oxacillinases families found in *A. baumannii* isolates are as follows: OXA-51 (chromosomally encoded), OXA-23, OXA-24/40, OXA-58, OXA-143 and OXA-235.

The most widespread resistance mechanisms among *A. baumannii* worldwide areOXA-23-like carbapenemases [16,17]. The location of the genes encoding these enzymes in *A. baumannii* relates to mobile genetic elements (i.e., transposons and insertion sequences, IS) [18,19].

CHDLs are enzymes with relatively weak activity that hydrolyze carbapenems with low efficiency. An occurrence of IS, additional nucleotide sequences upstream of *bla*_OXA_-like genes, promotes the expression of these genes [18]. The most prevalent IS among *A. baumannii* genomes are: IS*Aba1*, IS*Aba2*, IS*Aba3* or IS*18*, and they occur along with transposases genes and could be transferred between *A. baumannii* strain genomes [20]. IS*Aba1* is the most common IS among *A. baumannii* isolates and occurs in association with *bla*_OXA-23_, *bla*_OXA-51_ and *bla*_OXA-58_ genes. The genome of these bacteria also contains genetic structures covering important resistance genes—transposons (Tn). Three transposons have been described as related with *bla*_OXA-23_—Tn2006, Tn2007 and Tn2008. The structure of transposons is based on the presence of a resistance gene flanked by insertion sequences, e.g., in Tn2006, the *bla*_OXA-23_ gene is flanked by two copies of the insertion sequence IS*Aba1*, which are located in opposite directions [21].

The aim of this study was to determine the occurrence of *bla*_OXA-23_, *bla*_OXA-40_ and *bla*_OXA-51_ genes among *A. baumannii* strains susceptible to one of the carbapenems (meropenem) and to examine the occurrence of IS*Aba1* upstream of the *bla*_OXA-23_ gene among *bla*_OXA-23_-positive *A. baumannii* clinical isolates with their present impact on the carbapenem-resistance phenotype.

## 2. Materials and Methods

### 2.1. Bacterial Isolates Origin and Their Selection Criteria

In total, 35 *A. baumannii* clinical isolates, susceptible to meropenem, and 53 carbapenem-resistant (both imipenem and meropenem) *bla*_OXA-23_-positive *A. baumannii* were included in this study.

The species identification of the tested strains was determined using a MALDI-TOF MS instrument (Bruker, Mannheim, Germany) and MALDI Biotyper software (version 4.2.28), according to the manufacturer’s instructions. In order to ensure intra-laboratory quality control of identification during investigation, the bacterial test standard (BTS, Bruker, Mannheim, Germany) was used. For all the studied strains, an identification coefficient of ≥2.0 was obtained, which indicates a reliable assignment to the species.

Initially, all the tested strains were also checked for the presence of clinically important carbapenemase genes (types NDM, VIM, KPC, IMP, OXA-181 and OXA-48) using a commercially available kit (eazyplex^®^ SuperBug complete, AmplexDiagnostics GmbH, Gars-Bahnhof, Germany), and their results were negative for each isolate. Strain selection was set to include only isolates with a carbapenem resistance mechanism resulting at most from the presence of OXA-like enzymes. Therefore, the criteria for including isolates in this study were at most the presence of oxacillinases as the only one possible among the already known broad-spectrum beta-lactamases.

The isolates were stored in Brain Heart Infusion (Becton, Dickinson and Company, Sparks, MD, USA) with an addition of 15% glycerol (Sigma-Aldrich, St. Louis, MO, USA) at −80 °C. Before testing, they were grown on MacConkey Agar (Becton, Dickinson and Company, Sparks, MD, USA) at 37 °C for 24 h and re-passaged.

The *A. baumannii* DSMZ 102930 strain, purchased from Deutsche Sammlung von Mikroorganismen und Zellkulturen, Germany, served as the carbapenemase-positive control isolate, while the *A. baumannii* DSMZ 30008 isolate served as the carbapenemase-negative control strain. *Escherichia coli* ATCC 25922 (American Type Culture Collection); *P. aeruginosa* ATCC 27853 and both *A. baumannii* DSMZ isolates were also applied for the quality control of the applied methods.

### 2.2. Antimicrobial Susceptibility Testing

Antimicrobial susceptibility testing (AST) of the studied isolates was performed in duplicate using a BD Phoenix™ M50 instrument (Becton, Dickinson and Company, Sparks, MD, USA) using BD Phoenix™ NMIC-402 panels (Becton, Dickinson and Company, Sparks, MD, USA). The first AST was carried out as part of a routine laboratory diagnostic scheme and the second for research purposes, obtaining the same results each time.

Briefly, a dedicated AST indicator and 25 µL of 0.5 McFarland bacterial suspension were added into Mueller–Hinton broth (Becton, Dickinson and Company, Sparks, MD, USA). These suspensions were subsequently added to panels that were placed in a BD Phoenix™ M50 instrument, and the results were obtained automatically after less than 18 h. They were interpreted according to the European Committee on Antimicrobial Susceptibility Testing recommendation v. 13.0 (EUCAST) [22]. Quality control of AST was ensured by using the reference strains *P. aeruginosa* ATCC 27853 and *E. coli* ATCC 25922, also according to EUCAST recommendations.

The classification of the resistance phenotypes of the strains was as follows: multidrug-resistant (MDR)—strains resistant to at least three of the tested antimicrobial agents belonging to separate antibiotic groups; extensively drug-resistant (XDR)—strains resistant to all the tested antimicrobial agents except for two antimicrobial groups; pan-drug-resistant (PDR)—strains resistant to all the tested antimicrobials.

### 2.3. DNA Extraction

The tested and control *A. baumannii* strains were initially plated on MacConkey Agar (Becton, Dickinson and Company, Sparks, MD, USA) for 24 h at 37 °C and harvested afterwards for DNA isolation. DNA samples from the investigated strains were isolated using the kit for genomic DNA purification from various sources—Genomic Mini kit (A&A Biotechnology, Gdansk, Poland) according to the manufacturer’s protocol. DNA was stored at −20 °C before the testing.

### 2.4. PCR Analysis

#### 2.4.1. bla_OXA_ Gene Detection and High-Resolution Melting Analysis

Real-time PCR parameters and primers characterized in Table 1 were used to detect *bla*_OXA_ genes. The reactions were performed in a LightCycler 480 II thermal cycler (Roche, Basel, Switzerland). The final volume of each reaction was 20 µL, each consisting of 5 µL of molecular biology grade water (EurX, Gdansk, Poland), 5 µL (0.25 µM/reaction) of each primer as previously described [19,23] (Genomed, Warsaw, Poland), 4 µL of 5× HOT FIREPOL^®^ EvaGreen^®^ Mix (SolisBiodyne, Tartu, Estonia) and 1 µL of DNA of the strains included in this study. The following real-time PCR conditions were established: one cycle at 95 °C for 12 min, followed by 40 cycles at 95 °C for 15 s, annealing temperature at 58 °C for 20 s, and the final elongation with data collection at 72 °C for 20 s. Finally, the melting curve of the DNA amplification products was determined (95 °C for 5 s and 60 °C for 1 min, data acquisition at 0.11 °C increments between 60 °C and 97 °C) to confirm the specificity of the detected genes.

The data collection was enabled continuously at each increment during the temperature change (high-resolution melting variant). All the described real-time PCR procedures were initially performed as a standard PCR to confirm the specificity of the results and the product size confirmation using their standard electrophoretic separation in agarose gels (an applied procedure as described below).

#### 2.4.2. ISAba1-bla_OXA-23_ Gene Detection

A PCR assay for the detection of IS*Aba1*-*bla*_OXA-23_ was performed using the primers shown above (Table 1). The final volume of the PCR was 25 µL. The following reagents (all Promega, Walldorf, Germany) were used: MgCl_2_—2.5 mM; dNTPs—1 mM; polymerase GoTaq—1 U/reaction; primers—200 pM; 1.5× concentrated polymerase buffer and 2 µL of a DNA template. An amplification of the target gene was performed using a Mastercycler^®^Pro (Eppendorf, Hamburg, Germany). The reaction was performed according to the following conditions: one cycle at 94 °C for 5 min, followed by 30 cycles at 94 °C for 25 s, 54 °C for 40 s and 72 °C for 50 s with a final elongation at 72 °C for 6 min. The PCR reaction products were separated by electrophoresis on 1.5% agarose gel (Bio-Rad, Feldkirchen, Germany) in a 1× concentrated Tris-Boric Acid-EDTA (TBE, Bio-Rad, Feldkirchen, Germany) running buffer at 9 V/cm for 1 h in a MINI SUB^TM^ DNA CELL (Bio-Rad, Feldkirchen, Germany) device.

### 2.5. Data Analysis

Statistical analysis was performed using the Statistica™ 13.3 (TIBCO Software Inc., Palo Alto, CA, USA) program. Pearson’s chi-squared test and Fisher’s exact test were carried out to assess whether the differences in the strains’ origins and whether the changes in antibiotic resistance profiles were statistically significant. *p*-value of ≤0.05 was considered as statistically significant.

χ contingency coefficient values were used to investigate the strength of correlation for particular features’ coexistence and the strains’ origins (*p* ≤ 0.05). An interpretation of χ contingency coefficient correlation test was as follows: for χ: 0—no relationship; ±0.01–±0.19—none or insignificant relationship; ±0.2–±0.29—a weak relationship; ±0.3–±0.39—a moderate relationship; ±0.4–±0.69—a strong relationship; ±0.7 or higher—a very strong relationship.

## 3. Results

### 3.1. Bacterial Strains Origin

All of the tested strains were isolated between 2017 and 2023 from various patients (one strain per patient only) hospitalized at the University Hospital No. 1 in Bydgoszcz, Poland. The isolates were derived mainly from Anesthesiology and the Intensive Care Unit (ICU), and of them, 22.9% (*n* = 8) and 79.2% (*n* = 42) were susceptible and resistant to carbapenems, respectively. Table 2 and Table 3 show the detailed origin of *A. baumannii* clinical isolates used in this study.

**Figure 1:**
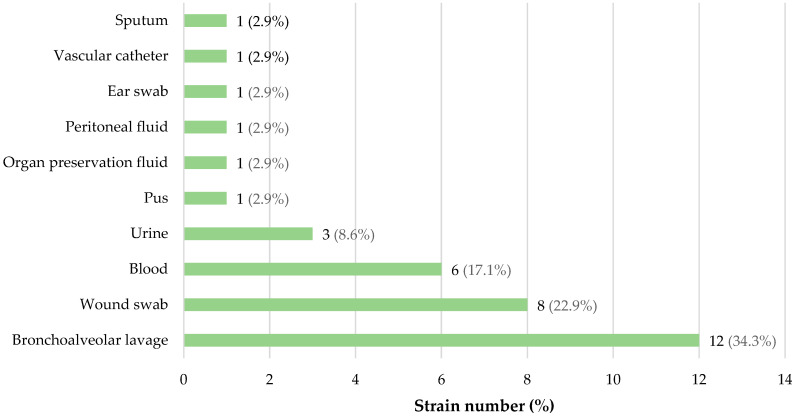
The origin of meropenem-susceptible *A. baumannii* strains used in this study, with respect to clinical specimen type (*n* = 35).

**Figure 2:**
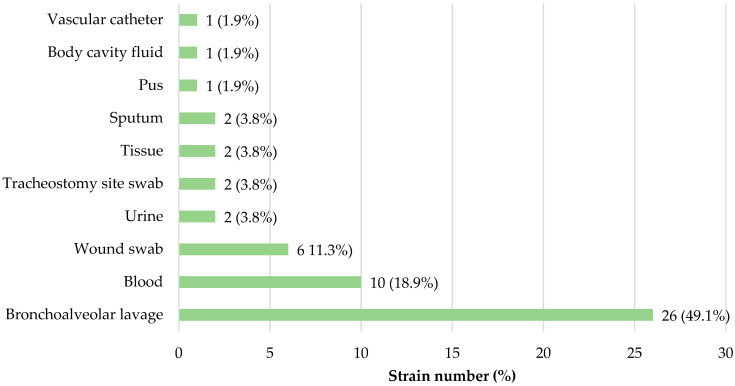
The detailed origin of *bla*_OXA-23_-positive *A. baumannii* strains used in this study, with respect to clinical specimen type (*n* = 53).

Figure 1 and Figure 2 show the detailed origin of *A. baumannii* strains used in this study with respect to clinical specimen type. Apart from four strains derived from a tracheostomy swab, an ear swab and organ preservation fluid, regardless of group belonging and strain characteristics, all the remaining bacterial strains were isolated from various types of infections, mainly from the cases of respiratory tract infections, wound swabs and blood samples.

### 3.2. Bacterial Strains Antimicrobial Susceptibility

Table 4 shows the antimicrobial susceptibility testing results among 35 meropenem-susceptible *A. baumannii* isolates. All of the strains, resistant simultaneously to quinolones and trimethoprim/sulfamethoxazole (*n* = 12, 34.3%), were also classified as multidrug-resistant (MDR) isolates. The majority of these strains were susceptible to colistin (*n* = 33, 94.3%). Nearly half (*n* = 16, 45.7%) of the isolates were susceptible with increased exposure to ciprofloxacin.

Antimicrobial susceptibility testing for carbapenem-resistant *bla*_OXA-23_-positive *A. baumannii* isolates also revealed the majority of isolates susceptible to colistin (*n* = 43, 86.8%). All of the tested isolates were resistant to carbapenems and quinolones (Table 5). All of the studied isolates were classified as extensively drug-resistant (XDR), and among these, nine (25.7%) isolates were pan-drug-resistant (PDR). Some of the strains were susceptible to amikacin and tobramycin exclusively—one (1.9%) and five strains (9.4%), respectively (Table 5).

A very strong relationship (χ = 0.768, *p* < 0.05) was found between the occurrence of MDR *A. baumannii* isolates and the presence of the *bla*_OXA-23_ gene. Meanwhile, there was no statistical relationship between colistin resistance and the presence of the *bla*_OXA-23_ gene (*p* > 0.05).

### 3.3. Presence of bla_OXA_ Genes among Meropenem-Susceptible A. baumannii Isolates

In total, 35 *A. baumannii* strains were tested for the presence of *bla*_OXA_ genes. Among these bacteria, 100% of the isolates carried the *bla*_OXA-51_ gene. The *bla*_OXA-23_ and *bla*_OXA-40_ genes were detected only in two (5.7%) and three (8.6%) isolates, respectively. IS*Aba1* fragments were detected upstream of *bla*_OXA-23_ in both isolates. Table 6 shows the drug susceptibility of *A. baumannii* isolates with confirmed *bla*_OXA_ gene presence. Two of the isolates were susceptible to imipenem. In addition, four of the studied strains were MDR.

### 3.4. Presence of ISAba1-bla_OXA-23_ among Carbapenem-Resistant A. baumannii Isolates (n = 53)

IS*Aba1* were altogether detected upstream of *bla*_OXA-23_ genes in 52 (98.1%) isolates. In the remaining *A. baumannii* strains, a PCR product with a different product length was detected. The only negative isolate for IS*Aba1*-*bla*_OXA-23_ with a product length of more than 962 bp was classified as XDR and was also susceptible to colistin. This mentioned *A. baumannii* isolate was isolated from bronchoalveolar lavage collected from an ICU patient.

A moderate relationship (χ = 0.526, *p* < 0.05) was found between multidrug resistance and strains’ origins—the ones derived from ICU patients.

## 4. Discussion

Infections caused by multidrug-resistant bacteria are an alarming problem worldwide [24,25], although the last few decades have been associated with a great development in medicine. In developed countries, access to healthcare systems is evident, but this is associated with an excessive drug uptake, use of biomaterials and invasive procedures that often cause nosocomial infections [26,27].

At the beginning of antibiotic therapy implementation, it was unpredictable that 70 years later, bacteria resistant to all treatment options would emerge [28]. In 2017, the World Health Organization published a list of priority pathogens for research and the development of new antibiotics, in which it was emphasized that carbapenem-resistant *A. baumannii* strains are a critical pathogen [29]. However, the understanding of the molecular basis of bacterial antimicrobial drug resistance is of fundamental importance in terms of their elimination purpose. Therefore, we aimed to decipher the carbapenem-resistant determinants resulting from insertion sequence presence among MDR *A. baumannii* clinical strains.

*A. baumannii* forms biofilms on both biotic and abiotic surfaces, facilitating survival on medical devices, hospital surfaces, etc. [6]. This is one of the reasons why these bacteria cause a number of VAP infections. In our study, most of the *A. baumannii* isolates (*n* = 38, 43.2%) were derived from specimens collected from the respiratory tract—bronchoalveolar lavage. Other researchers have also demonstrated the significant importance of *A. baumannii* strains on the development of respiratory tract infection, emphasizing VAP. Bandić-Pavlović et al. (2020) showed that 23.7% (*n* = 23) of *A. baumannii* isolates are associated with VAP infections, and 87.0 % (*n* = 20) among them belong to XDR strains [30].

Antimicrobial susceptibility testing among meropenem-susceptible isolates included in this present study shows that most of the antibiotics tested still had in vitro activity against *A. baumannii*, but even among those isolates, 12 (34.3%) were MDR and 16 (45.7%) were susceptible with increased exposure to ciprofloxacin. It shows that among these isolates, a choice of the appropriate therapy is also difficult. The hospital environment favors the persistence of MDR, often carbapenem-resistant strains [4,14,31].

Meanwhile, a global threat is the spread of carbapenem-resistant isolates among patients in hospitals. In this study, all the carbapenem-resistant isolates were classified as XDR, including recognition of nine (25.7%) of them as PDR isolates. The occurrence of carbapenem resistance is often associated with resistance to other antibiotic groups. All (*n* = 53, 100%) of the carbapenem-resistant *bla*_OXA-23_-positive *A. baumannii* strains included in this study were additionally resistant to quinolones. Moreover, other researchers have also shown that ciprofloxacin and levofloxacin combination is not a good choice for a treatment due to widespread resistance to fluoroquinolones among *A. baumannii* representatives [32].

The last-resort therapy with colistin is often the only treatment option for *A. baumannii* infections, yet, in our study, 11 (20.8%) strains were resistant to this antimicrobial also. Other studies have also shown worrying results regarding the emergence of colistin-resistant *A. baumannii* strains [33]. Almutairi et al. (2022) showed, as an actual necessity, the in vitro synergistic effect of using colistin with other antimicrobials, e.g., with vancomycin, rifamycin or beta-lactam, which is a quite surprising, however interesting and promising observation [34].

The focus of our study was carbapenemases, which are the most prevalent among *A. baumannii* bacteria worldwide: *bla*_OXA-23_, *bla*_OXA-40_ and *bla*_OXA-51_. OXA-like enzymes are considered relatively weak enzymatic carbapenem hydrolyzers. Therefore, in our study, meropenem-susceptible isolates of *A. baumannii* were also tested for the presence of *bla*_OXA_-like genes. In all of the tested strains, *bla*_OXA-51_ genes were present. This gene is an intrinsic representative of oxacillinases, localized in the chromosomal genome as has been previously described [35]. This gene has also been successfully used as a genetic marker for *A. baumannii* identification in a previous study [36]. *bla*_OXA-23_ and *bla*_OXA-40_ genes were not detected among a number of isolates (*n* = 30, 85.7%) in this study, with only two (5.7%) and three (8.6%) *A. baumannii* strains being positive for *bla*_OXA-23_ and *bla*_OXA-40_ genes, respectively. Three of these isolates were imipenem-resistant but two of them were surprisingly susceptible to both carbapenems. In both *bla*_OXA-23_-positive isolates, IS*Aba1* was detected. Noteworthily, one IS*Aba1*-*bla*_OXA-23_-positive isolate was also susceptible to imipenem. These results indicate the necessity of investigating the presence of various resistance mechanisms among *A. baumannii* or different expression levels among IS*Aba1*-*bla*_OXA-23_ genes for the phenotypic emergence of carbapenem resistance.

Analyzing isolates sensitive to meropenem, the authors identified isolates that presented the *bla*_OXA-40_ gene and the IS*Aba*1-*bla*_OXA-23_ set of genotypes associated with resistance to meropenem. This interesting fact could be elucidated by the investigation of possible differences in the mentioned gene sequences or expression levels for these genes between sensitive and resistant isolates. Hence, the suggested approach could explain the fact that even though these bacteria had genes associated with resistance, they were sensitive to the mentioned carbapenem. Therefore, in the next research step, the authors are planning to perform the DNA sequencing of the mentioned genes for the selected strains and to determine their relative expression levels. Through real-time PCR assays of the resistance genes found in isolates sensitive to meropenem, a comparison of them with those found in resistant strains should be possible. This could shed more light on the problem, providing more robust and obvious observations and conclusions.

The authors of this research paid attention the most to the strain selection and made an effort to choose non-repeatable strains for this analysis, e.g., only one strain per patient, different antimicrobial susceptibility profiles (if possible), different patient locations at the time of specimen collection and relatively long time periods between the strains’ isolations. However, we cannot be sure that the isolates are unique in molecular meaning and it would be beneficial to perform clonality testing, using multilocus sequence typing and comparative whole-genome sequencing in the ongoing research. In addition, it is planned to detect the molecular basis of resistance to antibiotics other than carbapenems.

Other researchers have shown that additional genetic elements, such as ISs, are needed to enhance the ability to hydrolyze carbapenems due to ISs acting as promoters of *bla*_OXA_-like gene expression. Several types of IS have been described so far, with IS*Aba1*, IS*Aba2*, IS*Aba3*, IS*Aba4*, IS*Aba9*, IS*Aba10*, IS*18* and IS*Aba825* among them, and IS*Aba1* as one of the most common in *A. baumannii* [21]. The presence of IS*Aba1* among *bla*_OXA-23_-positive isolates is also rather common. This has been also confirmed in our study, showing 98.1% (*n* = 52) of isolates carried IS*Aba1* upstream of the *bla*_OXA-23_ gene. These results are in consistence with some other research studies. Słoczyńska et al. (2021) showed the presence of IS*Aba1*-*bla*_OXA-23_ among 100% (*n* = 39) of the studied strains [19], while in 2015, Bahador et al. (2015) showed the occurrence of IS*Aba1* upstream of the *bla*_OXA-23_ gene in 67% (*n* = 34) of isolates only [37]. In this present study, for one isolate, an IS*Aba1* upstream *bla*_OXA-23_ gene was found but with a different product length. More research, e.g., DNA sequencing is needed to clarify the sequence of this particular product, but it is possible that there are more IS*Aba1* repeats in the genome of this particular isolate or that some genetic insertion within gene sequence appeared. Another limitation of this study was that the whole genome sequencing of the strains studied was not conducted to better characterize the isolates.

*A. baumannii* is one of the major nosocomial pathogens, causing severe infections in critically ill patients, hospitalized mostly within ICUs. Carbapenem-resistant *A. baumannii-*involved infections showed a significant association with length of ICU stay, increased treatment costs and antibiotic use [38]. Other studies have shown that *Acinetobacter* spp. infections represent 7.9% of VAP in the ICUs [39]. In this study, we showed that the isolation of *A. baumannii* from ICU had a moderate relationship with multidrug resistance among the strains of this species. Moreover, the resistance to carbapenems has been shown to also be associated with resistance to other treatment options. This was also described by Konca et al. (2021) in their study, showing *A. baumannii* clinical isolates resistant to all the treatment options, except for colistin and trimethoprim/sulfamethoxazole [40].

The characterization of isolates and epidemiological studies are also very important to track the transmission of *A. baumannii* between patients, especially in intensive care units. In this aspect, education, control and compliance with hygiene rules by staff are extremely important. Therefore, the comprehensive understanding of the molecular basis of carbapenem resistance is of great importance.

## 5. Conclusions

Carbapenem resistance among *A. baumannii* strains is a complex problem but is most often associated with the presence of CHDL, especially in the presence of IS. The clinical implications of these findings include the fact that the isolation of *bla*_OXA_-positive *A. baumannii* strains does not exclude completely the possibility of using carbapenems (or at least meropenem) in the treatment of infections caused by some such strains. Furthermore, in some cases, carbapenems with laboratory-proven in vitro activity should be considered active drugs against strains, regardless of the presence of insertion sequences in their DNA. However, in the case of empirical therapy for infections caused by strains with a high risk of oxacillinases presence, some other antimicrobial options should be considered safer (e.g., combination with aminoglycosides or colistin) and used successfully.

Although among the meropenem-susceptible isolates, only *bla*_OXA-51_ genes are identified, we show that it is important to monitor the susceptibility of *A. baumannii* isolates to antimicrobial agents and to study the molecular basis of resistance mechanisms, especially among endemic outbreaks. Of note, within *A. baumannii* isolates, the different expression levels of the IS*Aba1*-*bla*_OXA-23_ genes may influence the phenotypic manifestation and emergence of carbapenem resistance. Moreover, the same antimicrobial susceptibility pattern between the strains may result from a combination of different molecular determinants. The presence of IS*Aba1* among *bla*_OXA-23_-positive isolates is rather common. Although carbapenem-resistant *A. baumannii* clinical strains from Poland have a similar antimicrobial resistance profile as worldwide, this study demonstrated the need to investigate the presence of various bases and modes of these particular resistance mechanisms.

## Figures and Tables

**Table 1:** Sequences of primers applied for *bla*_OXA_ and IS*Aba1*-*bla*_OXA-23_ gene detection and PCR parameters used in the study.

Gene Detected	Primer Sequences 5′→3′	Tm (°C)	Annealing Temperature (°C)	Product Length (bp)	References
*bla*_OXA-40_	F: GGTTAGTTGGCCCCCTTAA	51.8	58	246	[23]
	R: AGTTGAGCGAAAAGGGGATT	49.7			
*bla*_OXA-23_	F: GATCGGATTGGAGAACCAGA	50.3	58	501	[23]
	R: ATTTCTGACCGCATTTCCAT	47.7			
*bla*_OXA-51_	F: TAATGCTTTGATCGGCCTTG	49.7	58	353	[23]
	R: TGGATTGCACTTCATCTTGG	49.7			
IS*Aba1*-*bla*_OXA-23_	F: AATCACAAGCATGATGAGCG	49.7	54	962	[19]
	R: CATTTCTGACCGCATTTCCAT	50.5			

**Table 2:** The detailed origin of meropenem-susceptible *A. baumannii* clinical strains used in this study (clinics/departments) (*n* = 35).

Clinic/Department	Strain Number (%)
Department of Anesthesiology and Intensive Care	8 (22.8%)
Clinical Unit of Anesthesiology and Intensive Care with Cardiac Anesthesiology Division	7 (20.0%)
Department of Cardiology	3 (8.6%)
Department of Neurology	3 (8.6%)
Department of General, Oncologic and Pediatric Urology	2 (5.7%)
Department of Liver and General Surgery	2 (5.7%)
Department of Nephrology, Hypertension and Internal Medicine	2 (5.7%)
Department of Otolaryngology and Laryngological Oncology with Audiology and Phoniatrics Unit	2 (5.7%)
Department of Dermatology, Sexually Transmitted Diseases and Immunodermatology	1 (2.9%)
Department of Emergency Medicine	1 (2.9%)
Department of Endocrinology and Diabetology	1 (2.9%)
Department of Orthopedics and Traumatology	1 (2.9%)
Department of Transplantation and General Surgery	1 (2.9%)
Chronic Wound Care	1 (2.9%)

**Table 3:** The origin of all *bla*_OXA-23_-positive *A. baumannii* clinical strains used in this study (clinics/departments) (*n* = 53).

Clinic/Department	Strain Number (%)
Department of Anesthesiology and Intensive Care	42 (79.2%)
Department of Endocrinology and Diabetology	3 (5.7%)
Department of Cardiology	2 (3.8%)
Department of Geriatrics	2 (3.8%)
Department of Liver and General Surgery	2 (3.8%)
Department of Cardiac Surgery	1 (1.9%)
Department of Rehabilitation	1 (1.9%)

**Table 4:** Antimicrobial susceptibility of *A. baumannii* meropenem-susceptible isolates (*n* = 35).

Number (%) of Strains			
Antimicrobial	Susceptible	Susceptible, Increased Exposure	Resistant
IMP	30 (85.7)	2 (5.7)	3 (8.6)
MEM	35 (100)	0	0
TOB	25 (71.4)	0	10 (28.6)
AMK	28 (80.0)	1 (2.9)	6 (17.1)
CIP	7 (20.0)	16 (45.7)	12 (34.3)
LEV	23 (65.7)	0	12 (34.3)
SXT	22 (62.9)	0	13 (37.1)
COL	33 (94.3)	0	2 (5.7)

IPM—Imipenem, MEM—Meropenem, TOB—Tobramycin, AMK—Amikacin, CIP—Ciprofloxacin, LEV—Levofloxacin, SXT—Trimethoprim/sulfamethoxazole, COL—Colistin.

**Table 5:** Antimicrobial susceptibility of carbapenem-resistant *bla*_OXA-23_-positive *A. baumannii* (*n* = 53).

Number (%) of Strains			
Antimicrobial	Susceptible	Susceptible, Increased Exposure	Resistant
IMP	0	0	53 (100)
MEM	0	0	53 (100)
TOB	5 (9.4)	0	48 (90.6)
AMK	1 (1.9)	0	52 (98.1)
CIP	0	0	53 (100)
LEV	0	0	53 (100)
SXT	0	2 (3.7)	51 (96.2)
COL	42 (79.2)	0	11 (20.8)

IPM—Imipenem, MEM—Meropenem, TOB—Tobramycin, AMK—Amikacin, CIP—Ciprofloxacin, LEV—Levofloxacin, SXT—Trimethoprim/sulfamethoxazole, COL—Colistin.

**Table 6:** Antimicrobial susceptibility among meropenem-susceptible *A. baumannii* isolates carrying *bla*_OXA_ genes (n = 5).

Specimen Origin	Unit	Gene Detected	IS*Aba1*	IMP	MEM	TOB	AMK	CIP	LEV	SXT	COL
Bronchoalveolar lavage	ICU	*bla*_OXA-23_	+	R	S	R	S	R	R	R	S
Wound swab	ICU	*bla*_OXA-23_	+	S	S	R	S	R	R	R	S
Pus	ICU	*bla*_OXA-40_	n/a	R	S	S	S	R	R	R	R
Urine	ICU	*bla*_OXA-40_	n/a	S	S	S	S	I	S	S	S
Vascular catheter	NEU	*bla*_OXA-40_	n/a	R	S	R	S	R	R	R	S

S—susceptible, I—susceptible with increased exposure, R—resistant, ICU—Anesthesiology and Intensive Care Unit, NEU—Department of Neurology, IPM—Imipenem, MEM—Meropenem, TOB—Tobramycin, AMK—Amikacin, CIP—Ciprofloxacin, LEV—Levofloxacin, SXT—Trimethoprim/sulfamethoxazole, COL—Colistin, +—positive result, n/a—not applicable.

## Data Availability

The original contributions presented in the study are included in the article, further inquiries can be directed to the corresponding author.
